# Multimodal Imaging in a Case of Fovea Plana Associated with Situs Inversus of the Optic Disc

**DOI:** 10.4274/tjo.galenos..2020.98415

**Published:** 2020-06-27

**Authors:** Berrak Şekeryapan Gediz, Mehmet Ali Şekeroğlu

**Affiliations:** 1 University of Health Sciences Turkey, Ankara Ulucanlar Eye Training and Research Hospital, Clinic of Ophthalmology, Ankara, Turkey

**Keywords:** Fovea plana, situs inversus of the optic disc, optic disc hypoplasia, tilted optic disc, multimodal imaging

## Abstract

Fovea plana is a congenital condition characterized by anatomic absence of the foveal pit. It may be isolated or associated with congenital ocular anomalies. In this report, we present a case of fovea plana associated with situs inversus of the optic disc, optic disc hypoplasia, tilted optic disc, and prepapillary vascular loop and with best corrected visual acuity of 20/32. The aim of this report is to demonstrate the coexistence of very rare multiple optic disc anomalies and fovea plana, and also to emphasize that the use of multimodal imaging methods facilitates the identification of rare anomalies.

## Introduction

Fovea plana, previously called foveal hypoplasia, is a condition characterized by the anatomic absence of the foveal pit. It may be isolated or associated with diseases such as albinism, microphthalmia, and achromatopsia. Fovea plana is usually bilateral, and visual acuity varies depending on accompanying pathologies.^1^ It has been reported that fovea plana may affect 3% of children, even those with normal visual acuity.^2^

Situs inversus of the optic disc is a rare congenital anomaly characterized by the abnormal course of vessels emerging from the optic disc.^3,4^ It may be associated with tilted optic disc and optic disc hypoplasia.^5^ In this report, we present a case of fovea plana associated with multiple optic disc anomalies consisting of situs inversus of the optic disc, optic disc hypoplasia, tilted optic disc, and prepapillary vascular loop, with multimodal imaging findings.

## Case Report

**Figure 1:**
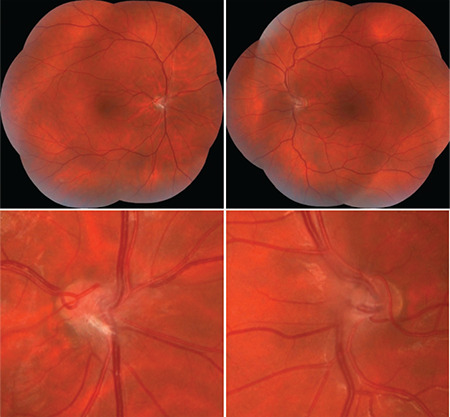
Colored fundus photographs of both eyes (upper panels) and magnified color photographs of both optic discs (lower panels). The colored fundus photographs show the retinal vessels emerge perpendicularly from the optic disc and are dilated, straight, and initially extend nasally before changing course to the temporal direction, especially in the left eye. The optic disc images demonstrate that both optic discs are hypoplastic and tilted, accompanied by gliotic tissue and prepapillary vascular loop, and vessels emerge nasally from the optic disc

**Figure 2:**
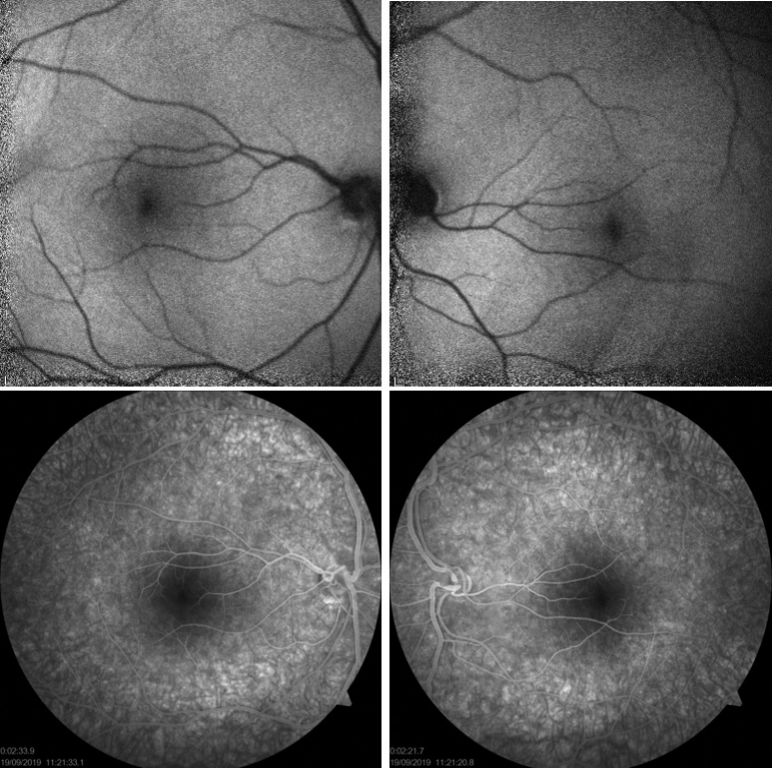
Fundus autofluorescence images of both eyes (upper panels) and fundus fluorescein angiography images of both eyes (lower panels). All images demonstrate hypoplastic and tilted optic discs and small foveal avascular zone

**Figure 3:**
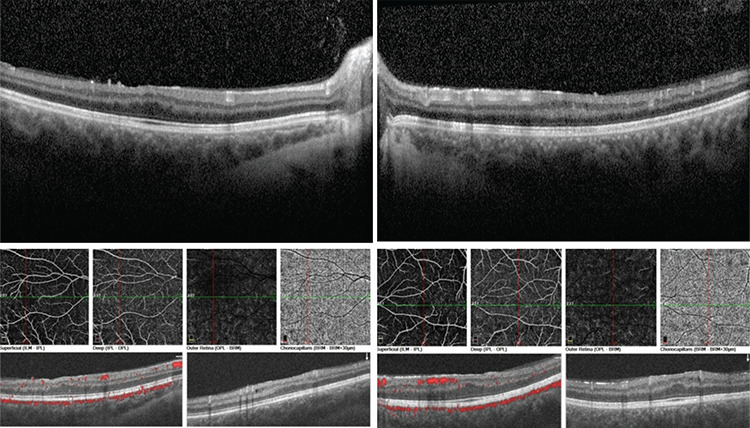
Optic coherence tomography images of both eyes (upper panels) and optic coherence tomography angiography images of both eyes (lower panels). Optic coherence tomography cross-sections passing through fovea demonstrate absence of the foveal pit and continuity of the internal retinal layers through the fovea. The optic coherence tomography angiography images also demonstrate absence of the foveal avascular zone in the superficial and deep capillary plexuses

A 25-year-old man presented with a long history of blurred vision. The patient had no known diseases or trauma history and his best corrected visual acuity was 20/32 with -0.50 D, -0.75 D x 180° in both eyes. Intraocular pressure was 12 mmHg in the right eye and 13 mmHg in the left eye, and anterior segment examination was normal. Fundus examination revealed that both optic discs were hypoplastic and tilted with accompanying gliotic tissue and prepapillary vascular loop; the vessels emerged perpendicularly, dilated, and straight from the optic disc and initially extended nasally before turning toward the temporal direction (Figure 1). Fundus fluorescein angiography revealed hypoplastic, tilted optic discs and small foveal avascular zone (Figure 2). Similar results were observed in fundus autofluorescence images (Figure 2). Optic coherence tomography (OCT) revealed absent foveal pit and continuity of the inner retinal layers through the fovea in both eyes (Figure 3). Central foveal thickness was 313 µm in the right and 312 µm in the left eye. Peripapillary retinal nerve fiber layer thickness measured with OCT was 39 µm in both eyes. OCT angiography (OCTA) demonstrated absence of the foveal avascular zone in both the superficial and deep capillary plexuses in both eyes (Figure 3). Axial length measured with A-mode ultrasound was 24.71 mm in the right and 24.72 mm in the left eye. The patient was diagnosed with fovea plana accompanied by multiple optic disc anomalies. The results of cranial magnetic resonance angiography to diagnose potential concomitant intracranial vascular pathologies were normal.

## Discussion

Situs inversus of the optic disc is a congenital embryonic anomaly characterized by blood vessels initially emerging nasally from the optic disc before turning sharply temporally. It is believed to occur as a result of anomalous insertion of the optic stalk into the optic vesicle and dysversion of the optic disc. It can be associated with other optic disc pathologies, primarily tilted optic disc.^3,4,5^ It is reported to affect 5% of the normal population.^6^

As the use of OCT became common in daily practice, we gained a better understanding of the anatomic changes that take place in patients with fovea plana. Continuation of the inner retinal layers through the fovea result in increased central foveal thickness and absence of the foveal pit is in OCT cross-sections.^7^ In OCTA studies of fovea plana patients, it has been reported that no foveal avascular zone is evident in the superficial or deep capillary plexus.^8^ Fovea plana may be associated with conditions such as albinism, aniridia, retinopathy of prematurity, achromatopsia, microphthalmia, myopia, and incontinentia pigmenti.^1^ There are reports in the literature that patients with optic disc hypoplasia have shallower foveal pit and increased central retinal thickness compared to normal eyes.^9^ Small optic disc can also be observed in patients with fovea plana associated with albinism and achromatopsy.^10^ Although these findings suggest a correlation between fovea and optic disc development, the etiopathogenesis of this association is not clear. In our case, fovea plana was observed with optic disc hypoplasia as well as findings of situs inversus, tilted disc, and prepapillary vascular loop. With this report, we aimed to draw attention to a very rare case of fovea plana with largely preserved visual acuity despite the coexistence of multiple optic disc anomalies. We also aimed to emphasize that the use of multimodal imaging methods facilitates the identification of rare anomalies.
